# Chlorophyll Pigments and Their Synthetic Analogs

**DOI:** 10.1093/pcp/pcae094

**Published:** 2024-08-22

**Authors:** Hitoshi Tamiaki, Saki Kichishima

**Affiliations:** Graduate School of Life Sciences, Ritsumeikan University, Kusatsu, Shiga, 525-8577 Japan; Graduate School of Life Sciences, Ritsumeikan University, Kusatsu, Shiga, 525-8577 Japan

**Keywords:** Biosynthesis, Cyclic tetrapyrrole, Excitation energy transfer, Stereochemistry, Substitution effect, Visible absorption

## Abstract

Oxygenic phototrophs use chlorophylls (Chls) as photosynthetically active pigments. A variety of Chl molecules have been found in photosynthetic organisms, including green plants, algae and cyanobacteria. Here, we review their molecular structures with stereochemistry, occurrence in light-harvesting antennas and reaction centers, biosyntheses in the late stage, chemical stabilities and visible absorption maxima in diethyl ether. The observed maxima are comparable to those of semisynthetic Chl analogs, methyl pyropheophorbides, in dichloromethane. The effects of their peripheral substituents and core π-conjugation on the maxima of the monomeric states are discussed. Notably, the oxidation along the molecular *x*-axis in Chl-*a* produces its accessory pigments, Chls-*b*/*c*, and introduction of an electron-withdrawing formyl group along the *y*-axis perpendicular to the *x*-axis affords far-red light absorbing Chls-*d*/*f*.

## Introduction

Chlorophylls (Chls) are one class of the most important pigments in oxygenic phototrophs (Senge et al. 2014, Bryant and Canniffe 2018, Croce and van Amerongen 2020, Kondo and Shibata 2022, Simkin et al. 2022). Since its first recognition approximately 200 years ago, a variety of Chl pigments have been found from the photosynthetic organisms (Tamiaki et al. 2007, Björn et al. 2009, Chen and Blankenship 2011, Tamiaki and Kunieda 2011, Kobayashi et al. 2013, Li and Chen 2015, Buscemi et al. 2022, Tamiaki 2022). In the initial stage of photosynthesis, Chls absorb sunlight, harvest the visible light, transfer the excitation energy and induce an electron-transfer process to form a charge-separating state. The first three steps occur in light-harvesting (LH) antenna systems, whereas the last step is performed at the core of reaction center (RC) complexes. All the photosynthetic apparatuses are produced by specific interactions of Chls and other pigments with peptides. Most of their supramolecular structures have been revealed at an atomic level by X-ray crystallography and cryo-electron microscopy (EM) (Hofmann et al. 1996, Jordan et al. 2001, Umena et al. 2011, Oba and Tamiaki 2014, Kato et al. 2020, 2024, Hamaguchi et al. 2021, Gisriel et al. 2022, Nagao et al. 2022, You et al. 2023). Here, we review the molecular structures of naturally occurring Chls as well as their variety in the photosynthetic apparatuses, late-stage biosyntheses, chemical alteration and visible absorption spectra in a solution. The visible absorption spectra of their monomeric states are dependent on the molecular structures, which have been confirmed by the spectroscopic analysis of their synthetic analogs. Chls are one of porphyrinoids with intense visible absorption bands, whose maxima are affected by the π-conjugation degree of the core cyclic tetrapyrroles and the functional groups at the peripheral positions.

## Naturally Occurring Chls

### Chl-*a*

**Fig. 1:**
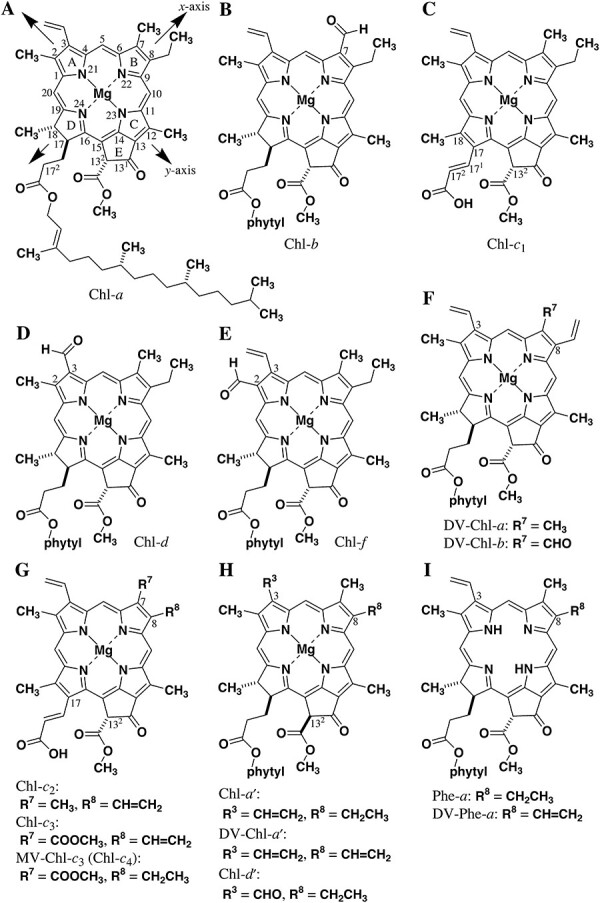
Molecular structures of (A–G) naturally occurring (DV-)Chls-*a*/*b*/*c*/*d*/*f*, (H) their prime forms and (I) their free bases in oxygenic phototrophs.

Chl-*a* is a representative chlorophyllous pigment, which was found from plants as the first major Chl pigment (Govindjee et al. 2024). Its molecular structure is based on four five-membered, nitrogen-containing pyrrole moieties connected with a methine group at the α-positions (see **Fig. 1A** and **Supplementary Fig. S1A**). Although the cyclic tetrapyrrole is π-conjugated, one of the composite pyrroles is hydrogenated at the β-positions to give a pyrroline ring. Another pyrrole ring is fused with a cyclopentane ring. The three pyrrole rings are named A-, B- and C-rings; the pyrroline ring is called D-ring; and the exo-five-membered ring is termed E-ring. The positions of 20 carbon atoms in the methine-linked tetrapyrrole are numbered clockwise from 1 at the α-position in the A-ring to 20. Similarly, the positions of the four nitrogen atoms in the cyclic tetrapyrrole are numbered from 21 in the A-ring to 24 in the D-ring. All the pyrrol(in)e rings are substituted at the β-positions. Methyl groups (-CH_3_) are introduced at the 2-, 7-, 12- and 18-positions; vinyl (-CH=CH_2_) and ethyl groups (-CH_2_CH_3_) are situated at the 3- and 8-positions, respectively; and a 2-(phytyloxycarbonyl)ethyl group (-CH_2_CH_2_COO-phytyl) is located at the 17-position. The E-ring possesses oxo (=O) and methoxycarbonyl groups (-COOCH_3_). The position numbers of carbon atoms on the E-ring are 13^1^ and 13^2^ for the 13-ethylene linker. Since the E-ring is produced in vivo by modifying a 2-carboxyethyl group at the 13-position of a biosynthetic precursor (Bryant et al. 2020), the methoxycarbonyl group is substituted at the 13^2^-position, but not the 15^1^-position. The numbering with superscript is applied for the other peripheral substituents. For example, the phytyloxycarbonyl group is substituted at the 17^2^-position.

The core of Chl-*a* contains three asymmetric carbon atoms at the 13^2^-, 17- and 18-positions. The stereochemistry is absolutely fixed as (13^2^*R*)-, (17*S*)- and (18*S*)-configurations (Fleming 1967, Senge et al. 2014). During the biosynthesis of Chl-*a* (Bryant et al. 2020), a (methoxycarbonyl)acetyl group (-COCH_2_COOCH_3_) at the 13-position is stereoselectively cyclized toward the 15-position to give an initial chiral carbon center at the 13^2^-position, i.e. the (13^2^*R*)-configuration (Tamiaki et al. 2016). Later, the C17=C18 double bond in a fully π-conjugated porphyrin system is dihydrogenated in a *trans*-manner. The diastereomeric hydrogenation of the (13^2^*R*)-enantiomer occurs at the C17-position from the same side of the (13^2^*R*)-COOCH_3_ to yield the (17*S*)-asymmetry with a less sterically demanding configuration where the 17-propionate residue is situated as the *anti*-form of the 13^2^-COOCH_3_. Concomitantly, the (18*S*)-stereochemistry is constructed through the *trans*-reduction to give a dihydrogenated porphyrin (= chlorin) diastereomer. The phytyl group is produced by three times reduction of a geranylgeranyl group with site- and stereoselective fashions to produce two more chiral centers, discussed later in the section ‘Chls with dehydrogenated phytyl groups’.

The central position of Chl-*a* bears a magnesium atom. The magnesium dication is bonded with four nitrogen atoms in the dianion species of the above cyclic tetrapyrrole prepared by deprotonation at the two N–H bonds. Although the neutral formula is shown in **Fig. 1**, the central Mg atom is always ligated with one more species to form a five-coordinated complex. As the core chlorin π-system is fairly planar, an additional ligand coordinates the Mg atom from either the upper or lower side. The axial ligation pulls the central Mg atom from the chlorin plane to form a pyramidal structure, causing the chirality at the Mg center due to structural differences around the four nitrogen atoms. The ligation from the same side as the 13^2^-COOCH_3_ group is called α-ligation (**Supplementary Fig. S1B**) and that from the opposite side is named β-ligation (Oba and Tamiaki 2005). The α- and β-ligated species are diastereomers. In a solution, the (de)ligation is so rapid that their differentiation could not be achieved by conventional spectroscopic analyses. Inside photosynthetic apparatuses, Chl-*a* molecules are axially ligated with any species including a histidyl imidazolyl residue and a water molecule. Because the ligation is completely fixed in peptides, the α- and β-ligated Chl-*a* species are visualized by X-ray crystallography and cryo-EM. The α-ligated Chl-*a* is observed more frequently than the β-ligated in photosynthetically active peptides analyzed by the above techniques (Balaban et al. 2002, Oba and Tamiaki 2002, 2014, Kawamoto et al. 2020). This α-ligation preference is consistent with the computational calculation results that the α-ligated species is more thermodynamically stable than the corresponding β-ligated diastereomer (Oba and Tamiaki 2002, 2005).

### Chl-*b* and Chl-*c*

Chls-*b* and *c* are minor pigments in Chl-*a*-dominant phototrophs and separated from major Chl-*a* by chromatographic techniques. The excitation energy of Chls-*b*/*c* is efficiently transferred to Chl-*a* in the LH antenna systems (see further). The formers, Chls-*b*/*c*, are thus called accessory pigments.

Chl-*b* is the 7-formyl (7-CHO) substituent of Chl-*a* (**Fig. 1B**). The transformation of the 7-methyl group of Chl-*a* or its precursor to the 7-formyl group is catalyzed by Chl-*a* oxygenase (CAO) (Oster et al. 2000). In the enzymatic process, the 7-methyl group is initially oxidized to the 7-hydroxymethyl group (7-CH_2_OH) followed by direct dehydrogenation to the 7-CHO or one more hydroxylation at the 7^1^-position and successive dehydration of the resulting 7-dihydroxymethyl group [7-CH(OH)_2_]. It is noted that the dehydration of a hydrated aldehyde readily proceeds in a solution to give the aldehyde: RCH(OH)_2_ → RCHO + H_2_O.

Chl-*c* is a series of related pigments, Chls-*c*_1_, *c*_2_ and *c*_3_ (see section ‘Chls-*c* and their related pigments’). Typically, Chl-*c*_1_ is the 17,17^1^,17^2^,18-tetradehydrogenated and dephytylated derivative of Chl-*a* (**Fig. 1C**). All the Chls-*c* are fully π-conjugated porphyrins possessing a *trans*-2-carboxyethenyl group (-CH=CHCOOH) at the 17-position, whereas Chls-*a*/*b* carry a chlorin π-system with a phytyl-esterified 2-carboxyethyl group at the same position. The former and latter 17-substitutes are also called acrylate and propionate residues, respectively. The biosynthetic pathway of Chls-*c* is described in section ‘Chls-*c* and their related pigments’. Both Chls-*b*/*c* as the accessory pigments are Chl-*a* analogs oxidized along the molecular *x*-axis, the N22–N24 diagonal line in **Fig. 1A**.

### Chl-*d* and Chl-*f*

Chl-*d* is a unique Chl pigment. It was first found as the 3-formylated Chl-*a* (**Fig. 1D**) in red alga approximately 80 years ago (Manning and Strain 1943). Later, it was proposed to be the artifact of Chl-*a* during its isolation process. It was reported in 1996 that *Acaryochloris marina*, a cyanobacterium, almost exclusively produced Chl-*d* with a faint amount of Chl-*a* (Miyashita et al. 1996). Chl-*d* has been widely recognized as one of the photosynthetically active pigments. In some other cyanobacteria producing Chl-*a* exclusively under standard cultivation conditions, Chl-*d* is observed with far-red light illumination (Kühl et al. 2020, Gisriel et al. 2022). Under far-red light photoacclimation (FaRLiP), Chl-*f* is produced as the 2-formylated Chl-*a* (**Fig. 1E**) (Chen et al. 2010). Chls-*d*/*f* prepared through FaRLiP are at most 10% of the original Chl-*a* amount. The photoinduced oxidation of the 2-methyl to formyl group is catalyzed by Chl-*f* synthase (Ho et al. 2016) in a similar manner as the 7-CH_3_ to CHO by CAO (Chen et al. 2023), but the biosynthetic transformation of the 3-vinyl to formyl group has not been revealed perfectly. The latter oxidation at the 3-substituent might be requisite for molecular oxygen and cysteinyl sulfanyl group (-SH) (Chen and Blankenship 2011, Fukusumi et al. 2012, Loughlin et al. 2013). It is noteworthy that both Chls-*d*/*f* absorbing far-red light are Chl-*a* analogs oxidized along the molecular *y*-axis (the N21–N23 diagonal line in **Fig. 1A**) perpendicular to the *x*-axis.

After the initial detection of Chl-*d*, a Chl species with specific visible absorption bands measured for the faint samples from natural staffs was named as Chl-*e* in 1940s (Li and Chen 2015). Its molecular structure has not been determined yet due to its less availability from any phototrophs. In 2010, a new Chl pigment (not Chl-*e*) was discovered from a cyanobacterium, *Halomicronema hongdechloris*, and its molecular structure (2-formylated Chl-*a*) was fully characterized by obtaining its large amount via the successful FaRLiP cultivation (Chen et al. 2010). To avoid confusion, the new pigment was named Chl-*f*. At present, Chl-*e* is not still confirmed as a photosynthetically active pigment and might be an artifact of Chl-*a* (Sorimachi et al. 2015).

### Divinyl-Chls

The 8-vinyl derivatives of Chls-*a*/*b* are observed in marine cyanobacteria, *Prochlococcus* (see R^7^ = CH_3_/CHO in **Fig. 1F**) (Partensky et al. 1993). Because these Chl pigments possess two vinyl groups at the 3- and 8-positions, they are named divinyl(DV)-Chls-*a*/*b*. Some researchers call them Chls-*a*_2_/*b*_2_, which are not accepted by Chl society. To differentiate the standard Chls-*a*/*b* from DV-Chls-*a*/*b*, Chls-*a*/*b* are sometimes called monovinyl(MV)-Chls-*a*/*b*. During the biosynthesis of Chl-*a*, the 8-vinyl group is dihydrogenated to the 8-ethyl group by divinyl reductase (DVR) (Nagata et al. 2005). *Prochlococcus* lacks DVR to produce DV-Chls-*a*/*b* exclusively. Since DV-Chls-*a*/*b* absorb blue light more efficiently than Chls-*a*/*b* (Ting et al. 2002), *Prochlococcus* species are blue light acclimated cyanobacteria. In addition, artificial DVR-lacking mutants of oxygenic phototrophs produced DV-Chls-*a*/*b* predominantly (Nagata et al. 2005).

### Chls-*c* and their related pigments

As mentioned in section ‘Chl-*b* and Chl-*c*’, Chls-*c* are fully π-conjugated porphyrins with an acrylate residue at the 17-position. The 1,2-disubstituted ethylene always takes a *trans*-configuration that is more thermodynamically stable than the *cis*-form. The C17^1^=C17^2^ double bond is situated on the same side of the C17=C18 double bond to give a *cisoid* conformation in the planar 1,3-butadiene moiety (see **Fig. 1C, G**). The acrylate residue in the *transoid* prepared by the rotation around the C17–C17^1^ single bond of the *cisoid* interacts with the 13^2^-methoxycarbonyl group more crowdingly than that in the *cisoid*. This preference is confirmed by molecular modeling calculation (Mizoguchi et al. 2009). Chls-*c* carry only one asymmetric carbon at the 13^2^-position. Naturally occurring Chls-*c* are enantiomerically pure and absolutely (13^2^*R*)-stereoisomers (Helfrich et al. 2003), which were characterized by chiral high-performance liquid chromatography (HPLC) (Mizoguchi et al. 2010, 2011).

Chl-*c*_2_ is the 8-vinyl analog of Chl-*c*_1_ (see R^7^ = CH_3_ and R^8^ = CH=CH_2_ in **Fig. 1G**). The didehydrogenation at the 8-ethyl group in Chl-*c*_1_ to Chl-*c*_2_ is the same as that of Chl-*a* to DV-Chl-*a*. From the situation, DV-Chl-*a* is occasionally called Chl-*a*_2_ (see section ‘DV-Chls’), but nobody accepts Chl-*a*_1_ as the original Chl-*a*. Chl-*c*_3_ is the 7-methoxycarbonyl derivative of Chl-*c*_2_ (R^7^ = COOCH_3_ and R^8^ = CH=CH_2_ in **Fig. 1G**), both of which bear 3,8-DV groups. In 2012, the 8-ethyl analog of Chl-*c*_3_ (R^7^ = COOCH_3_ and R^8^ = CH_2_CH_3_ in **Fig. 1G**) was found in a phytoplankton (haptophyte) producing Chl-*c*_3_, *Emiliania huxleyi* (Álvarez et al. 2012). The analog can be called MV-Chl-*c*_3_, and we here propose it to name Chl-*c*_4_.

**Fig. 2:**
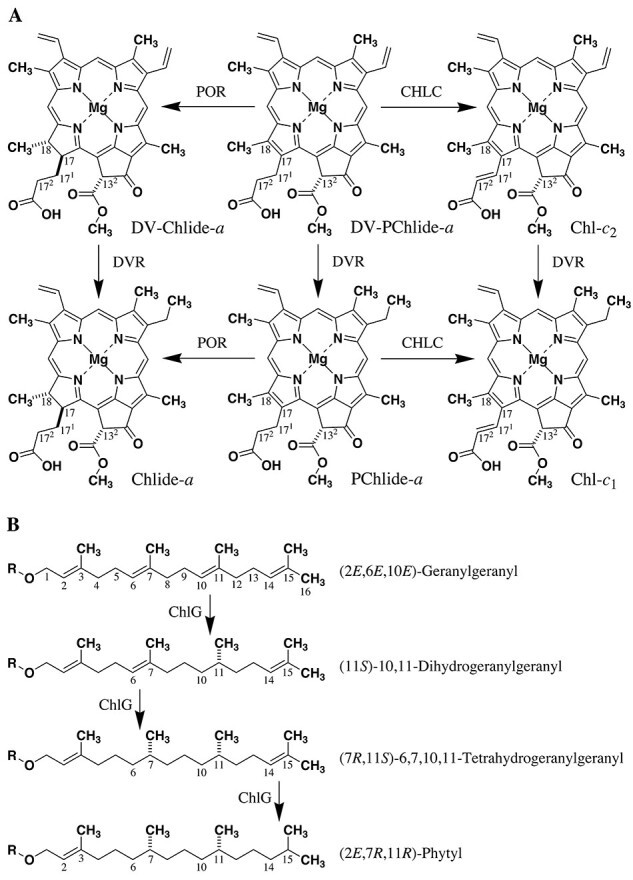
Biosynthetic pathways of (A) (DV-)PChlide-*a* to Chl-*c*_1_/*c*_2_ and (DV-)Chlide-*a* and (B) in sequential hydrogenation of geranylgeranyl to phytyl group by ChlG: ROH = Chlides or diphosphoric (pyrophosphoric) acid.

The 17-acrylate residue is specific to Chls-*c*, whereas the other Chls bear the 17-propionate residues. Although an acrylate residue is found in some other natural porphyrinoids including heme-*d*_1_ (Xu et al. 2016, Klünemann et al. 2019), its biosynthetic pathway had not been unresolved. Very recently, the didehydrogenation of a propionate to acrylate residue at the 17-position was revealed (Jiang et al. 2023, Jinkerson et al. 2024). The oxidation of the 17-CH_2_CH_2_ to 17-CH=CH moiety is catalyzed by a 2-oxoglutarate-dependent dioxygenase, Chl-*c* synthase (CHLC). The reaction occurs via the direct didehydrogenation of the 17-propionate residue or its hydroxylation at the 17^1^-position followed by the dehydration of the resulting benzyl-type alcohol [17-CH_2_CH_2_COOH → CH(OH)CH_2_COOH → CH=CHCOOH] (Wu et al. 2016). Protochlorophyllide-*a* (PChlide-*a*) is one of the Chl-*a* biosynthetic precursors and possesses a fully π-conjugated porphyrin skeleton and a propionate residue at the 17-position (see the lower, central drawing in **Fig. 2A**) (Shiozaki et al. 2023). The oxidation of PChlide-*a* at the 17^1^- and 17^2^-positions by CHLC produces Chl-*c*_1_ (**Fig. 2A**, lower, right), whereas its reduction at the 17- and 18-positions by PChlide oxidoreductase (POR) affords chlorophyllide-*a* (Chlide-*a*, see **Fig. 2A**, lower, left) (Muraki et al. 2010, Zhang et al. 2019, Silva and Cheng 2022, Pesara et al. 2024). Similarly, DV-PChlide-*a* is oxidized by CHLC to give Chl-*c*_2_ and reduced by POR to yield DV-Chlide-*a* (**Fig. 2A**, upper) (Shiozaki et al. 2023). The 8-vinyl groups in DV-PChlide-*a*, DV-Chlide-*a* and Chl-*c*_2_ are reduced by DVR to produce PChlide-*a*, Chlide-*a* and Chl-*c*_1_, respectively.

Chl-*c*_3_ and Chl-*c*_4_ (MV-Chl-*c*_3_) should be biosynthesized through the transformation of a methyl to methoxycarbonyl group at the 7-position, but the related enzyme has not been identified yet. The oxidation of the 7-methyl to carboxy group might be catalyzed by a CAO-like enzyme (see section ‘Chl-*b* and Chl-*c*’), and the resulting carboxylic acid would be esterified to the 7-methoxycarbonyl group. The 17^1^,17^2^-dihydrogenated form of Chl-*c*_3_ possessing the 17-propionate residue would be a biosynthetic precursor of Chl-*c*_3_, and isolated from a tropical green alga, *Micromonas pusilla* (Álvarez et al. 2013): the pigment was formerly called Chl-*c*_CS-170_. The pigment possessing the 7-methoxycarbonyl group might be produced from DV-PChlide-*b* bearing the 7-formyl group. Chl-*c*_4_ would be biosynthesized from its 17^1^,17^2^-dihydrogenated form, which has not been detected in any natural phototrophs, or produced by the DVR-reduction of Chl-*c*_3_. DV-PChlide-*a* is a key precursor of Chls as mentioned above, and might be a photosynthetically active pigment in some green algae (Ricketts 1970, Álvarez et al. 2013): it is sometimes called magnesium 2,4-divinylpheoporphyrin-*a*_5_ monomethyl ester.

Chls-*c*_2_ esterified at the 17-acrylate residue were isolated from *E. huxleyi* and another haptophyte (Garrido et al. 2000, Zapata et al. 2001, Tamiaki et al. 2007). The esterifying groups are based on monogalactosyldiacylglycerol. It is unclear whether these esters function as photosynthetic pigments or not. The photosynthetically active Chls-*a*/*b*/*d*/*f* are usually esterified with phytol. The esterification is performed in the final stage of their biosynthetic pathways (Bryant et al. 2020). Free acids, Chlides-*a*/*b*/*d*/*f* react with phytyl diphosphate in the catalytic action of chlorophyll synthase (ChlG) to give Chls-*a*/*b*/*d*/*f*. Alternatively, similar enzymatic reactions with geranylgeranyl diphosphate by ChlG and successive reduction of the geranylgeranyl to phytyl group by geranylgeranyl reductase (ChlP) produce the above phytylated Chls (vide infra). ChlG is inactive for the esterification of free carboxylic acids in Chls-*c* and PChlides bearing fully π-conjugated porphyrin cores.

### (13^2^*S*)-Epimers of Chls

In oxygenic phototrophs, RC complexes are observed in two-type photochemical systems, photosystem I (PSI) and PSII. The cores of the RC complexes are primarily composed of Chl-*a*, DV-Chl-*a* or Chl-*d*, but no (DV-)Chl-*b*, Chls-*c* and Chl-*f*. The charge-separating core in PSI-RC contains the 13^2^-epimer of the composite Chl pigments, called prime forms, (DV-)Chls-*a*′ and Chl-*d*′ with the (13^2^*S*)-stereochemistry (see **Fig. 1H**). The prime forms interact with its normal forms to produce the dimeric species in primary electron donors, P700 with (DV-)Chls-*a*/*a*′ (Kobayashi et al. 1988, Jordan et al. 2001, Komatsu et al. 2016) and P740 with Chls-*d*/*d*′ (Hamaguchi et al. 2021) giving redmost absorption bands at 700 and 740 nm, respectively. The epimerization at the chiral 13^2^-positions from the (*R*) to (*S*) configuration would occur enzymatically, but no related epimerase has been determined. Since the stereochemistry at the 13^2^-position in the β-keto-ester moiety (13-COCHCOOCH_3_) can be readily inverted in a solution (Hynninen et al. 1979, Watanabe et al. 1987, Mazaki et al. 1992), the epimerization might occur under non-enzymatically base-assisted conditions.

The benzylic C13^2^-H was weakly acidic due to the presence of two neighboring electron-withdrawing carbonyl groups. Under basic conditions, the proton is abstracted to give its enolate species, providing an epimeric mixture through the reverse protonation from the opposite side of the 17-propionate residue. As mentioned in the biosynthetic hydrogenation of the D-ring, the (13^2^*R*) form is less sterically crowding and more thermodynamically stable than its epimeric prime species with the (13^2^*S*)-stereochemistry. The epimerization equilibrium thus affords a 3:1 to 4:1 mixture of normal and prime forms (Watanabe et al. 1987, Ogasawara et al. 2020b). The epimerization kinetics were examined by HPLC. The stereochemical inversion of Chl-*a* with the 7-methyl group in a basic solution occurred slightly more slowly than that of Chl-*b* with the 7-formyl group (Tanaka et al. 2022). The acceleration in the epimerization of Chl-*b* over Chl-*a* is ascribable to the electronegativity of the 7-substituents, i.e. the electron-withdrawing CHO group enhances the acidity of the C13^2^-H in Chl-*b* to give the formation of the enolate more readily than Chl-*a*. It is noted that no epimerization under basic conditions occurred for Chl-*a* homologs possessing a methylene or ethylene group between the C13^2^–COOCH_3_ bond as well as a Chl-*a* derivative where the 13^2^-COOCH_3_ was replaced with a propyl group due to the less acidity at their C13^2^-H (Ogasawara and Tamiaki 2015, Ogasawara et al. 2015, 2020a).

Free acrylate Chl-*c*_1_ in an aqueous solution was epimerized more slowly than the corresponding free propionate PChlide-*a* (Shiozaki et al. 2023). The chemical stability of Chl-*c*_1_ over PChlide-*a* would be explained by the following two factors. (1) Since an acrylate anion (-CH=CH–COO^−^) is less weakly basic than an propionate anion (-CH_2_CH_2_COO^−^), the former acrylate reacts with the C13^2^-H to give the enolate species less readily than the latter propionate. (2) The 13^2^-deprotonation in Chl-*c*_1_ can occur intermolecularly, but not intramolecularly, due to the far situation of the 17^2^-COO^−^ with the 13^2^-H, whereas the 17^2^-COO^−^ in PChlide-*a* can quickly deprotonate the 13^2^-H in a molecule to produce the enolate species (see **Supplementary Fig. S2**). The intramolecular interaction of the 17-propionate residue with the 13^2^-H is comparable to that with the C18-atom in the reduction pathway of the C17=C18 double bond by dark-operative POR (see **Supplementary Fig. S3**) (Muraki et al. 2010).

### Free bases of Chls

In the charge-separating cores of PSII-RC complexes, metal-free Chl pigments, called pheophytins (Phes), are primary electron acceptors. (DV-)Chls-*a*-producing phototrophs contain (DV-)Phes-*a* (see **Fig. 1I**) (Zouni et al. 2001, Umena et al. 2011, Komatsu et al. 2016), whereas cyanobacteria producing mainly Chl-*d* utilize Phe-*a* as the primary electron acceptor in PSII-RC, not Phe-*d* (Akiyama et al. 2001). The removal of a magnesium ion at the central position in (DV-)Chls-*a* could occur by dechelatase (Shimoda et al. 2016, Dey et al. 2022), which has not been identified in phototrophs yet. The pheophytinization of Chls proceeds in an aqueous acidic solution so easily (Joslyn and Mackinney 1938, Saga and Tamiaki 2012, Sadaoka et al. 2013, Saga et al. 2013) that the formation of (DV-)Phes-*a* might be non-enzymatically performed in a solution. No appearance of Phe-*d* might be related to the fact that Chl-*d* possessing an electron-withdrawing formyl group at the 3-position is more tolerant to the acidic pheophytinization in a solution than Chl-*a* with the less electron-withdrawing 3-vinyl group (Hirai et al. 2009, Sato et al. 2024). It is noted that the above formation of Phes must be achieved by the pheophytinization of Chls because the insertion of a magnesium ion at the central position by a chelatase occurs at a relatively earlier stage in the biosynthesis of Chls (Bryant et al. 2020).

Although (DV-)Chls-*a* function as primary electron acceptors in PSI-RCs producing (DV-)Chls-*a* (Jordan et al. 2001), the primary electron acceptor in PSI-RC of *A. marina* producing Chl-*d* was found to be Phe-*a* (neither Chl-*d* nor Chl-*a*) from the cryo-EM analysis (Hamaguchi et al. 2021). In the specific cyanobacterium, primary electron acceptors in both the PSI-RC and PSII-RC are the same, Phe-*a*.

### Chls with dehydrogenated phytyl groups

In addition to the aforementioned prime forms (13^2^-epimerized Chls) and free base species (metal-free Phes), minor Chls esterified with different diterpenoids from a conventional phytyl group are observed in oxygenic phototrophs under specific conditions. In greening processes, geranylgeranylated, dihydrogeranylgeranylated and tetrahydrogeranylgeranylated Chls-*a*/*b* were observed as biosynthetic precursors to phytylated (hexahydrogeranylgeranylated) Chls-*a*/*b* (Nakamura et al. 2001, Tamiaki et al. 2017). With high-light illumination, such Chl-*a* precursors were isolated from a diatom (Mizoguchi et al. 2017), and also green light cultivation of green plants accumulated these Chl-*a*/*b* precursors (Karlický et al. 2021, Paper et al. 2022). The molecular structures of the diterpenoids were fully characterized by spectroscopic and chromatographic analyses as well as their comparison with the authentic samples (**Fig. 2B**) (Tamiaki et al. 2017). The sequential hydrogenation of a geranylgeranyl group by ChlP occurs at C10=C11, C6=C7 and C14=C15 double bonds (Tamiaki et al. 2017). It is interesting that the hydrogenation starts at the middle of four double bonds and the order is not one way from the C6=C7 to C14=C15 or its reverse. The stereoselective and regioselective hydrogenations are unique for biosynthetic pathways in Chls or diphosphates with geranylgeranyl to phytyl groups.

## Synthetic Chl Analogs

**Fig. 3:**
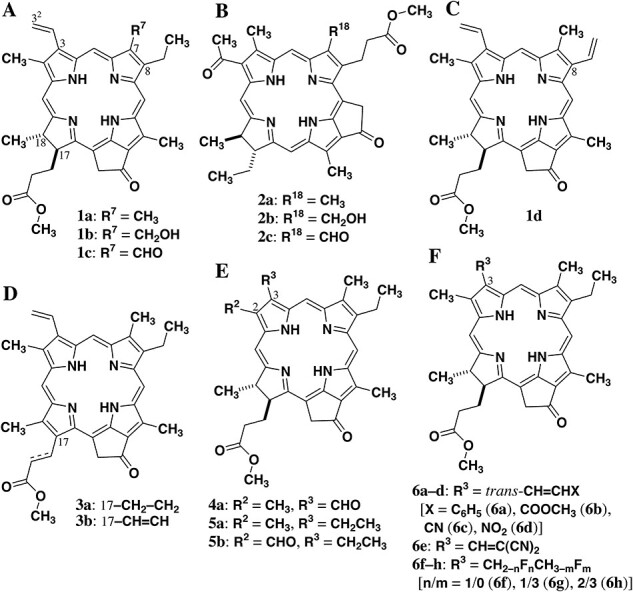
Molecular structures of synthetic Chl analogs, methyl pyropheophorbides **1**–**6**.

Chl-*a* was extracted from commercially available cyanobacterial cells producing only Chl-*a* as a chlorophyllous pigment. The crude extract was treated with an aqueous acidic solution (removal of the central Mg by pheophytinization), methanol containing sulfuric acid (exchange of the phytyl to methyl group by transesterification) and refluxing collidine (removal of the 13^2^-COOCH_3_ by pyrolysis) to give methyl pyropheophorbide-*a* (**1a**, see R^7^ = CH_3_ in **Fig. 3A**). The product was purified by silica gel column chromatography and recrystallization to afford the pure sample of **1a** (Smith et al. 1985, Ma and Dolphin 1996, Pandey et al. 1996, Tamiaki et al. 1998).

From commercially available spinach powders, a mixture of Chl-*a* (major) and Chl-*b* (minor) was extracted, because green plants including spinach utilized Chl-*b* as an accessory pigment (vide supra). Chl-*b* can be separated from Chl-*a* by chromatography, but the separation of the large amounts is tedious due to their similar molecular polarities. To solve the problem, the following procedures were developed (Oba et al. 1997, Tamiaki et al. 2000). Following the same chemical modification as in Chl-*a* to **1a** (vide supra), a mixture of Chls-*a*/*b* was transformed into **1a**/**c** (R^7^ = CH_3_/CHO in **Fig. 3A**) and treated with a mildly reducing reagent (*tert*-butylamine borane complex). The highly reactive 7-formyl group of **1c** was selectively reduced to the corresponding 7-hydroxymethyl group in **1b** (R^7^ = CH_2_OH in **Fig. 3A**). Since the obtained alcohol was more polar than intact **1a** bearing the 7-methyl group, the former **1b** was readily separated from the latter **1a** by simple silica gel column chromatography. With a large amount of chemically pure **1b** in hand, the primary alcohol was smoothly oxidized to the aldehyde, **1c**.

As a model of DV-Chl-*a*, 8-vinylated chlorins including **1d** (**Fig. 3C**) were prepared as follows (Kunieda et al. 2004). Since the C7=C8 double bond in a chlorin moiety was reactive, the treatment of an 8-ethyl-chlorin with osmium tetroxide and hydrogen sulfide afforded its 7,8-dihydroxy-adduct (see **Supplementary Fig. S4A**). The *cis*-diol was doubly dehydrated to give the corresponding 8-vinyl-chlorin. The 8-vinyl group as a peripheral substituent could be chemically modified to a variety of functional groups.

PChlide-*a* analogs possessing a fully π-conjugated porphyrin skeleton were prepared by didehydrogenation at the C17H–C18H moiety in a chlorin chromophore (Tamiaki et al. 1999). The selective oxidation of **1a** to the corresponding porphyrin was impossible. Its zinc metalation reduced the oxidation potential, so the desired oxidation by a strong oxidation reagent (2,3-dichloro-5,6-dicyano-1,4-benzoquinone, DDQ) readily occurred for the zinc complex of **1a**. After demetalation by the action of an acid, methyl pyroprotopheophorbide-*a* (**3a**; **Fig. 3D**) was obtained.

Chl-*c* analogs can be prepared by chemical modification of Chl-*c* extracted from commercially available diatom cells. The diatom produces both Chl-*c*_1_ (minor) and Chl-*c*_2_ (major) as Chl-*c* pigments, which must be separated. Similar to the separation of Chls-*a*/*b* mentioned above, the large-scale isolation of the pure Chl-*c* pigments is challenging because of their low solubilities in conventional organic solvents and slight difference in the polarity (the 8-ethyl *vs*. 8-vinyl groups). Alternatively, methyl pyropheophorbide-*c*_1_ (**3b**; **Fig. 3D**) was prepared from **1a** (Xu et al. 2016). The key step for the preparation of **3b** is based on the *cis*-dihydroxylation at the C17=C18 double bond of a porphyrin and the double dehydration of the resulting *cis*-diol (**Supplementary Fig. S4B**), which is comparable to the conversion of the 8-ethyl to 8-vinyl group (vide supra).

Methyl pyropheophorbide-*d* (**4a**; R^2^ = CH_3_ and R^3^ = CHO in **Fig. 3E**) bearing the 3-formyl group was easily synthesized by the oxidative cleavage of the 3-vinyl group in **1a** (Tamiaki et al. 1996). This availability is due to the reactive 3-vinyl group. By contrast, the less labile 2-methyl group is hard to be selectively modified by simple procedures. Methyl mesopyropheophorbide-*f* (**5b**; R^2^ = CHO and R^3^ = CH_2_CH_3_ in **Fig. 3E**) possessing the 2-formyl group was produced by multiple steps (Xu et al. 2014). In the preparation, the 3-vinyl group must be altered to the less reactive 3-ethyl group. The oxidation at the 2-methyl to formyl group is ascribable to the *cis*-dihydroxylation at the C2=C3 double bond and the single dehydration of the resulting *cis*-diol to give a mixture of 2^1^- and 3^1^-hydroxy products (**Supplementary Fig. S4C**). The 2-hydroxymethyl-chlorin was separated from the regioisomeric 3-(1-hydroxyethyl)chlorin and oxidized to the 2-formyl-chlorin. Zinc metalation of a chlorin is necessary for the selective dihydroxylation at the 2,3-positions, because the C7=C8 double bond is predominantly oxidized in the free base of a chlorin (vide supra).

The 3-formyl group is more chemically reactive than the 3-vinyl group. Aldehyde **4a** was transformed into **6a**–**d** (R^3^ = *trans*-CH=CHX in **Fig. 3F**) as the (*E*)-3^2^-monosubstituted form of **1a**, via Wittig or Henry reaction (**Supplementary Fig. S5**; Tamiaki and Kouraba 1997, Tamiaki et al. 2014). Knoevenagel reaction of **4a** (Tamiaki and Kouraba 1997) afforded **6e** with a β,β-disubstituted vinyl group at the 3-position (**Fig. 3F**). Methyl mesopyropheophorbides **6f**–**h** fluorinated at the 3-ethyl group of **5a** (**Fig. 3F**) were prepared through deoxy- or deoxofluorination at the 3^1^-position and trifluoromethylation of the 3-formyl group (**Supplementary Fig. S5**; Nishibori et al. 2024).

## Visible Absorption Maxima of Chls

### Naturally occurring Chls

As mentioned above, Chls-*b*/*c* are accessory pigments for a large amount of Chl-*a* in oxygenic phototrophs. These accessory pigments in photosynthetic organisms absorb blue to green light, which is intense in solar spectrum, in their Soret bands more efficiently than major Chl-*a* (see **Supplementary Fig. S6**). Since the site energies of Chls-*b*/*c* are higher than that of Chl-*a*, the former excitation energy is quickly and effectively transferred to the nearby Chl-*a*. The situation is primarily attributable to the optical properties of their Chl molecules despite the secondary effects of their environmental factors. Here, we discuss the visible absorption maxima (*λ*_max_) of the naturally occurring Chls and their synthetic analogs in organic solvents. Chls are magnesium complexes and dissolved in diethyl ether. In the diluted solutions, the central Mg atom of Chls is axially coordinated with the oxygen atom of a diethyl ether molecule to form pyramidal species (vide supra). The five-coordinated complexes are well-dispersed in diethyl ether solvent to be monomeric species.

In diethyl ether, Chl-*a* exhibits two intense absorption bands in a visible region. The visible absorption bands at shorter and longer wavelengths are called Soret and Q*y* bands, respectively. The Soret maximum [*λ*_max_(Soret)] is observed at 430 nm, while the Q*y* peak [*λ*_max_(Q*y*)] is situated at 661 nm (**Table 1**). Most oxygenic phototrophs absorb sunlight using these Soret and Q*y* bands. The Soret band is corresponding to the second excitation energy level (S_2_), whose state is internally and quickly converted to a lower excitation energy state (S_1_ state). The S_1_ state corresponds to the Q*y* maximum and is more stable with a longer lifetime compared with the S_2_ state. From the S_1_ state, excitation energy and electron are transferred in LH and RC apparatuses, respectively.

**Table 1:** Visible absorption maxima (*λ*_max_/nm) of naturally occurring Chls in diethyl ether

Compound	Soret	Q*y*	References
Chl-*a*	430	661	Tamiaki and Kunieda 2011
Chl-*b*	453	642	Tamiaki and Kunieda 2011
DV-Chl-*a*	436	660	Tamiaki and Kunieda 2011
PChlide-*a*	432	622	Tamiaki and Kunieda 2011
Chl-*c*_1_	444	626	Tamiaki and Kunieda 2011
Chl-*c*_2_	448	626	Tamiaki and Kunieda 2011
Chl-*d*	446	686	Tamiaki and Kunieda 2011
Chl-*f*	440	695	Li et al. 2012

**Fig. 4:**
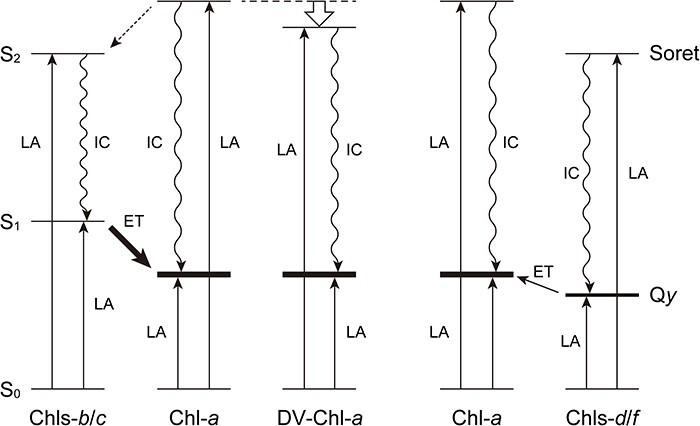
Schematic drawings of energy levels in Chls with light absorption (LA), internal conversion (IC) and excitation energy transfer (ET).

Chl-*b* with the 7-formyl group in diethyl ether shows a Soret maximum at 453 nm, which is a longer wavelength by 23 nm than that of Chl-*a* with the 7-methyl group. As a result, Chl-*b* can absorb blue light more efficiently than Chl-*a*. The presence of Chl-*b* in LH systems is useful for harvesting sunlight. The Q*y* maximum of 7-formylated Chl-*b* at 642 nm is shifted to a shorter wavelength by 19 nm than that of 7-methylated Chl-*a*. The former S_1_ state is higher in energy than the latter. The excitation energy of Chl-*b* can be transferred to its neighboring Chl-*a* (**Fig. 4**, left). The exothermic excitation energy transfer readily occurs in LH systems. Since the lifetimes of S_2_ states are much shorter than those of S_1_ states as mentioned above, the excitation energy transfer from the S_2_ state of Chl-*a* to that of Chl-*b* is usually negligible but possible under specific conditions (Gotze and Lokstein 2023).

Chls-*c* afford relative intense Soret and weak redmost (Q*y*) absorption bands, compared to those of Chl-*a*. The difference is owing to the fact that Chls-*c* are fully π-conjugated porphyrins and Chl-*a* is a dihydroporphyrin with a chlorin π-skeleton. In a diluted diethyl ether solution, Chls-*c* exhibit Soret and Q*y* maxima at longer and shorter wavelengths, respectively, than those of Chl-*a*: *λ*_max_(Soret) = 444/448 (Chls-*c*_1_/*c*_2_) > 430 nm (Chl-*a*) and *λ*_max_(Q*y*) = 626 (Chls-*c*_1_/*c*_2_) < 661 nm (Chl-*a*). This situation is the same as that observed for Chl-*b* mentioned above. Therefore, Chls-*c* function as accessory pigments for Chl-*a*.

The Soret maximum of DV-Chl-*a* with the 8-vinyl group at 436 nm in diethyl ether is bathochromically shifted from that of Chl-*a* with the 8-ethyl group (430 nm), although the former DV-Chl-*a* exhibits almost the same Q*y* maximum as the latter Chl-*a*: *λ*_max_(Q*y*) = 660 (DV-Chls-*a*) ≈ 661 nm (Chl-*a*). Similarly, as in Chls-*b*/*c*, DV-Chl-*a* absorbs blue light more efficiently than Chl-*a* (**Fig. 4**, center). Because the site energy of DV-Chl-*a* is comparable to that of Chl-*a*, the excitation energy is used for the following charge separation in the RC core without further modifications. Therefore, marine cyanobacteria producing DV-Chl-*a* as well as DV-Chl-*b* and DV-PChlide-*a* (Ralf and Repeta 1992) are well adapted to the blue-enriched environment (450–500 nm) of the deeper surface layers of the oligotrophic ocean without relying on resource-intensive pigments such as phycobilin (Ting et al. 2002, Hickman et al. 2010).

Both the Soret and Q*y* maxima of Chl-*d* with the 3-formyl group in diethyl ether are red-shifted from those of Chl-*a* with the 3-vinyl group: *λ*_max_(Soret/Q*y*) = 446/686 (Chl-*d*) > 430/661 nm (Chl-*a*). Chl-*d* absorbs blue and far-red lights more effectively than Chl-*a*. The light that is not blocked by the photosynthesis of the Chl-*a* organisms can still be used by the Chl-*d* organisms. As a result, cyanobacteria producing predominantly Chl-*d* would survive underneath the Chl-*a*-producing phototrophs, similar to the aforementioned DV-Chl-*a* species. Since the S_1_ state of Chl-*d* is lower in energy than that of Chl-*a*, the charge-separating and electron-transferring RC system must be tuned by altering the nearby peptides from the standard system using Chl-*a*. It is noted that both the primary electron acceptors in the PSI- and PSII-RC cores are identical to be Phe-*a*, but neither Chl-*d* nor Phe-*d* (vide supra).

Similar to Chl-*d*, Chl-*f* with the 2-formyl group exhibits the Soret and Q*y* maxima at longer wavelengths than Chl-*a* with the 2-methyl group: *λ*_max_(Soret/Q*y*) = 440/695 (Chl-*f*) > 430/661 nm (Chl-*a*) in diethyl ether. Especially, the Q*y* peak of Chl-*f* (695 nm) is shifted more bathochromically than that of Chl-*d* (686 nm). Under FaRLiP conditions, some cyanobacteria using Chl-*a* produce Chl-*f* as well as Chl-*d* as LH pigments (Gisriel 2024) because Chls-*d*/*f* can absorb far-red light above 700 nm. The S_1_ states of Chls-*d*/*f* are higher in the excitation energy level than that of Chl-*a*. The partial substitution of Chl-*a* with Chls-*d*/*f* in LH systems indicates that up-hill excitation energy transfer from the latter Chls-*d*/*f* to the former Chl-*a* must be operated (**Fig. 4**, right). The endothermic energy transfer is less effective but does occur slowly.

### Synthetic Chl analogs

To clarify the aforementioned effect of substituents in natural Chls on visible absorption maxima, the visible absorption spectra of synthetic Chl analogs, methyl pyropheophorbides as shown in **Fig. 3**, were measured in dichloromethane. Their spectra in a diluted solution provide an intense Soret band as well as a sharp Q*y* band, which are corresponding to the monomeric bands. Methyl pyropheophorbide-*a* (**1a**) as a Chl-*a* model exhibits Soret and Q*y* maxima at 414 and 667 nm, respectively (see **Table 2**). The observed former value (414 nm) is smaller than that of Chl-*a* in diethyl ether (430 nm), whereas the latter (667 nm) is larger than *λ*_max_(Q*y*) of Chl-*a* (661 nm). The shifts are explained by magnesium metalation at the central position of porphyrinoids (Taniguchi et al. 2022). Although the change of the solvents slightly affects the monomeric maxima, the 13^2^-methoxycarbonyl group and the 17-propionate residue hardly disturb the bands due to the lack of their direct π-conjugation with the core chlorin system.

**Table 2:** Visible absorption maxima (*λ*_max_/nm) of synthetic Chl analogs, methyl pyropheophorbides, in dichloromethane

Compound	Soret	Q*y*	References
**1a** (7-CH_3_; 2.47^a^)	414	667	Funakoshi and Tamiaki 2021
**1b** (7-CH_2_OH; 2.59^a^)	418	662	Funakoshi and Tamiaki 2021
**1c** (7-CHO; 2.87^a^)	440	656	Funakoshi and Tamiaki 2021
**2a** (18-CH_3_)	416	691	Funakoshi and Tamiaki 2021
**2b** (18-CH_2_OH)	422	687	Funakoshi and Tamiaki 2021
**2c** (18-CHO)	451	679	Funakoshi and Tamiaki 2021
**1a** (8-CH_2_CH_3_; 2.48^a^)	414	667	Xu et al. 2016
**1d** (8-CH=CH_2_; 2.79^a^)	422	667	Xu et al. 2016
**1a** (C17H–C18H/17-CH_2_CH_2_)	414	667	Xu et al. 2016
**3a** (C17=C18/17-CH_2_CH_2_)	418	640	Xu et al. 2016
**3b** (C17=C18/17-CH=CH)	436	653	Xu et al. 2016
**1a** (3-CH=CH_2_; 2.79^a^)	414	667	Xu et al. 2014
**4a** (3-CHO; 2.87^a^)	428	694	Xu et al. 2014
**5a** (2-CH_3_; 2.47^a^)	409	656	Xu et al. 2014
**5b** (2-CHO; 2.87^a^)	411	691	Xu et al. 2014

a Electronegativity of substituents (Inamoto and Masuda 1982).

Methyl pyropheophorbide-*b* (**1c**) as a Chl-*b* model shows Soret and Q*y* maxima at 440 and 656 nm, respectively, in dichloromethane. The former value (440 nm) is shifted to a longer wavelength than that of **1a** (414 nm), whereas the latter (656 nm) moves to a shorter wavelength than that of **1a** (667 nm). The bathochromic (+26 nm) and hypsochromic shifts (−11 nm) are comparable to those observed for Chl-*a* → Chl-*b* (+23 and −19 nm). The substitution effect of a methyl to formyl group at the 7-position in natural Chls on the absorption maxima is reproduced by the synthetic models. The introduction of a hydroxymethyl group at the 7-position in **1b** induces smaller shifts in *λ*_max_(Soret/Q*y*) from those of **1a**, compared with the 7-formylation of **1a** to **1c**. A group electronegativity (*χ*) enhances in the order of methyl, hydroxymethyl and formyl groups: *χ* = 2.47 (CH_3_) < 2.59 (CH_2_OH) < 2.87 (CHO) (Inamoto and Masuda 1982). An increase in the electron-withdrawing ability of the 7-substituent bathochromically shifts the Soret maxima and hypsochromically shifts the Q*y* maxima. Therefore, the oxidation of the 7-methyl group as in Chl-*a* to *b* is useful for the production of an accessory pigment.

By chemically modifying bacteriochlorophyll-*a*, chlorins bearing methyl (**2a**; Liu et al. 2008), hydroxymethyl (**2b**; Funakoshi and Tamiaki 2021) and formyl groups (**2c**; Funakoshi and Tamiaki 2021) as the R^18^-substituent shown in **Fig. 3B** were prepared. The substituents are situated at the opposite side of the pyrroline moiety in a chlorin chromophore, which is comparable to the 7-position of **1**. The substitution effect of the R^18^-group in **2a**–**c** on the Soret and Q*y* maxima is similar to that of the R^7^-group in **1a**–**c**: ∆*λ*_max_(Soret) and ∆*λ*_max_(Q*y*) = +6/+29 and −4/−8 nm for **2a** → **2b**/**2b** → **2c**  *vs*. +4/+22 and −5/−6 nm for **1a** → **1b**/**1b** → **1c**. The enhancement in the oxidation degree at the 7^1^-position moves Soret and Q*y* peaks to longer and shorter wavelengths, respectively, which is confirmed by the above two model systems.

The Soret and Q*y* maxima of methyl divinyl-pyropheophorbide-*a* (**1d**), which is a model of DV-Chl-*a*, are located at 422 and 667 nm, respectively. The *λ*_max_(Soret) value of **1d** with the 8-vinyl group is larger by 8 nm than that of **1a** with the 8-ethyl group (414 nm), but the *λ*_max_(Q*y*) value of the 3,8-divinyl form is identical to that of the 3-monovinyl form (667 nm). The red shift of Soret maxima and no shift of Q*y* maxima by the 8^1^,8^2^-didehydrogenation (8-ethyl to 8-vinyl) is comparable to the shifts by the transformation of natural Chl-*a* to DV-Chl-*a*. Substitution of an ethyl group at the 8-position with a vinyl group allows natural and synthetic chlorins to absorb blue light more efficiently and does not change their S_1_ states. Considering that a vinyl group is more electronegative than an ethyl group [*χ* = 2.79 (CH=CH_2_) > 2.48 (CH_2_CH_3_)], the 8-substitution effect on the Soret maxima is similar to the 7-substitution effect, but the former 8-substitution effect on the Q*y* maxima is different from the latter 7-substitution effect.

Methyl pyroprotopheophorbide-*a* (**3a**) possessing a fully π-conjugated porphyrin skeleton exhibits Soret and Q*y* maxima at 418 and 640 nm in dichloromethane, respectively. The 17,18-didehydrogenation of a chlorin chromophore in **1a** to **3a** induces the bathochromic and hypsochromic shifts of the Soret and Q*y* maxima, respectively: ∆*λ*_max_(Soret/Q*y*) = +4/−27 nm for **1a** → **3a**. This is typical of the electronic absorption bands in porphyrinoids with a different π-conjugation in the core system. A decrease in the π-conjugation degree of porphyrins to chlorins (dihydroporphyrins) and bacteriochlorins (dihydrochlorins) affords blue shifts of Soret bands and red shifts of Q*y* bands (Linnanto 2022). Further didehydrogenation in the 17-substituent of **3a** with a propionate residue to **3b** with an acrylate residue moves both the Soret and Q*y* maxima to longer wavelengths: ∆*λ*_max_(Soret/Q*y*) = +18/+13 nm for **3a** → **3b**. The bathochromic shifts are ascribable to the π-conjugation of the 17-acrylate residue with the porphyrin core. The aforementioned dehydrogenation effects of synthetic chlorins **1a**/**3a**/**3b** on Soret and Q*y* maxima are comparable to those of natural Chl-*a*/PChlide-*a*/Chl-*c*_1_: ∆*λ*_max_(Soret/Q*y*) = +2/−39 nm for Chl-*a* → PChlide-*a* and +12/+4 nm for PChlide-*a* → Chl-*c*_1_. Since the tetradehydrogenation of **1a** to **3b** moves the Soret maxima to a longer wavelength by 22 nm, **3b** absorbs blue light more efficiently than **1a**. Additionally, the transformation of **1a** into **3b** raises the energy level of the S_1_ state [∆*λ*_max_(Q*y*) = −14 nm], and the singlet excitation energy transfer from **3b** to **1a** is exothermic. These changes from **1a** to **3b** resemble those from Chl-*a* to Chl-*c*_1_: ∆*λ*_max_(Soret/Q*y*) = +14/−35 nm for Chl-*a* → Chl-*c*_1_. The function of Chl-*c*_1_ as the accessory pigment of Chl-*a* is thus confirmed by the model system.

The oxidative cleavage of a vinyl group at the 3-position of **1a** to the 3-formyl group of **4a** shifts both Soret and Q*y* maxima to longer wavelengths by 14 and 27 nm, respectively. Similarly, the oxidation of a methyl group at the 2-position of **5a** to the 2-formyl group of **5b** induced bathochromic shifts of both Soret and Q*y* maxima: ∆*λ*_max_(Soret/Q*y*) = +2/+35 nm. The electron-withdrawing effects are comparable to those in Chl-*a* to Chls-*d*/*f* bearing the 3/2-formyl groups: ∆*λ*_max_(Soret/Q*y*) = +16/+25 nm for Chl-*a* to *d* and +10/+34 nm for Chl-*a* to *f*. The model systems reproduce the effective absorption of blue and far-red lights in natural Chls-*d*/*f*. Since the shift in the Q*y* maxima by the formylation at the 2-position (+35 nm) is larger than that by the 3-formylation (+27 nm), Chl-*f* as the 2-formylated Chl-*a* absorbs far-red light more efficiently than Chl-*d* as the 3-formylated Chl-*a*: *λ*_max_(Q*y*) = 695 (Chl-*f*) > 686 nm (Chl-*d*).

To confirm the aforementioned 3-substitution effect, the visible absorption spectra of methyl 3^2^-substituted pyropheophorbides-*a*  **6a**–**e** were measured in dichloromethane. When a phenyl group is introduced at the 3-vinyl terminal of **1a** in a *trans*-manner, the resulting 3-*trans*-styryl substitute **6a** exhibits the Soret and Q*y* maxima at 420 and 673 nm, respectively, which are bathochromically shifted by 6 nm from those of 3^2^-unsubstituted **1a** (see **Table 3**). The observed shifts are ascribed to an elongation of the π-conjugation along the molecular *y*-axis and an increase in the electronegativity of a hydrogen atom (*χ* = 2.18) to a phenyl group (2.72). The 3^2^-substitutions of **1a** with methoxycarbonyl as in **6b** and cyano groups as in **6c** similarly move their Soret/Q*y* maxima to 421/684 and 420/689 nm, respectively. The 3^2^-nitration of **1a** to **6d** shifts its Soret and Q*y* maxima to much longer wavelengths: ∆*λ*_max_(Soret) = +14/+36 nm and ∆*λ*_max_(Q*y*) = +35 nm for **1a** → **6d**. The Q*y* maxima are correlated with the electron-withdrawing abilities of the 3^2^-substituents. An increase in their group electronegativities shifts the Q*y* maxima bathochromically: *λ*_max_(Q*y*) = 667 (**1a**; *χ* = 2.18 for the 3^2^-H) < 684 (**6c**; 2.83 for COOCH_3_) < 689 (**6d**; 3.21 for CN) < 702 nm (**6e**; 3.42 for NO_2_). The 3^2^,3^2^-dicyanation of **1a** to **6e** induces further red shifts of Soret and Q*y* maxima at 431/454 and 706 nm, respectively, which is due to the electronic effect mentioned above.

**Table 3:** Visible absorption maxima (*λ*_max_/nm) of methyl 3-substituted pyropheophorbides in dichloromethane

Compound	Soret	Q*y*	References
**1a** (3-CH=CH_2_; 2.18^a^)	414	667	Funakoshi and Tamiaki 2021
**6a** (3-CH=CHC_6_H_5_; 2.72^a^)	420	673	Tamiaki and Kouraba 1997
**6b** (3-CH=CHCOOCH_3_; 2.83^a^)	421	684	Tamiaki and Kouraba 1997
**6c** (3-CH=CHCN; 3.21^a^)	420	689	Tamiaki and Kouraba 1997
**6d** (3-CH=CHNO_2_; 3.42^a^)	428/450	702	Tamiaki et al. 2014
**6e** (3-CH=C(CN)_2_)	431/454	706	Tamiaki and Kouraba 1997
**5a** (3-CH_2_CH_3_)	409	656	Nishibori et al. 2024
**6f** (3-CHFCH_3_)	410	663	Nishibori et al. 2024
**6g** (3-CHFCF_3_)	411	671	Nishibori et al. 2024
**6h** (3-CF_2_CF_3_)	411	676	Nishibori et al. 2024

a Electronegativity of X-substituents in 3-*trans*-CH=CHX (Inamoto and Masuda 1982).

The 3^1^-fluorination of 3-ethylchlorin **5a** to **6f** moves the Soret peak to a slightly longer wavelength (409 → 410 nm) and largely red-shifts the Q*y* maximum (656 → 663 nm) (see **Table 3**). This shift in the Q*y* peaks is owing to the high electronegativity of a fluorine atom over a hydrogen atom. Since a 1-fluoroethyl group is more electron-withdrawing than an ethyl group, **6f** with the former group at the 3-position (3-CHFCH_3_) along the molecular *y*-axis exhibits the Q*y* maximum at a longer wavelength than that of **5a** with the latter group (3-CH_2_CH_3_). Similarly, as in the monofluorination of **5a** to **6f**, the 3^2^,3^2^,3^2^-trifluorination of **6f** to **6g** affords red shifts of Soret and Q*y* maxima by 1 and 8 nm, respectively. The bathochromic shifts are explained by an enhancement in the electronegativity from a methyl (*χ* = 2.47) to trifluoromethyl group (2.99). The perfluorination at the 3-ethyl group of **5a** to **6h** further moves the Soret and Q*y* maxima at 411 and 676 nm, respectively. The effect of the sequential fluorination of **5a** to **6f**–**h** on visible absorption maxima is ascribable to the strong electronegativity of a fluorine atom and consistent with the effect of the 3^2^-substitution of **1a** to **6a**–**e** (vide supra).

## Perspective

Molecular structures of major photosynthetically active Chls were identified, and the absolute configurations of their asymmetric carbon atoms were confirmed. Minor Chl species in oxygenic phototrophs have been almost characterized, but new Chl pigments might be present in nature. Novel Chl species might be found in phototrophs grown in specific environments including high or low temperature, high or low light intensity, limited illumination wavelength and lack or reduction of any culturing elements. During extraction and/or separation of such molecules, they might be altered due to their less chemical stabilities in solution, so these chemically labile Chls have not been identified yet. More careful investigation of chromophyta containing haptophyta, cryptophyta and dinophyta might lead to new Chls-*c* and their related pigments bearing a porphyrin π-skeleton, which would be photosynthetically active pigments or their biosynthetic precursors. Since 2-, 3- and 7-formylated Chl-*a* analogs were found as Chls-*f, d* and *b*, respectively, 8-formylated Chl-*a*, tentatively called Chl-*g*, is one of the candidates for new Chls, which is produced by the transformation of the 8-ethyl group in Chl-*a* or the 8-vinyl group in DV-Chl-*a*. Furthermore, Chl-*e* proposed from the specific visible absorption bands should be hunted in natural phototrophs.

Biosynthetic pathways from DV-PChlide-*a* to most Chls have been identified, but their reaction mechanisms have not fully revealed. Some of the biosynthetic enzymes have been characterized in atomic level by X-ray single crystallographic analysis technique. Cryo-EM as well as computer-assisted modeling (e.g. AlphaFold or simulation docking) would be helpful for revealing the mechanisms. Large-scale preparations of enzyme peptides and substrate pigments are important for the investigation. The biosynthesis of minor Chl species is less clarified, and especially, epimerase for the 13^2^-stereochemical inversion and dechelatase for the removal of the central magnesium atom should be identified.

Ultraviolet and visible absorption spectra of Chls in solution were reported by several groups, but certain data of their absorption (extinction) coefficients are limited. These values must be determined for the future investigation of LH and energy-transferring processes in photosynthesis. Although conventional down-hill (exothermic) excitation energy transfer has been well investigated by a variety of spectroscopic techniques, up-hill (endothermic) energy transfer should be more analyzed, whose mechanisms must be clarified. In addition, the visible absorption spectra of Chls should be reproduced by any theoretical manners, since present theoretical calculations including time-dependent density functional theory could not afford their precise spectra.

## Supplementary Material

pcae094_Supp

## Data Availability

Source data for tables are provided in the paper.
