# Supplementary material for: Chlorophyll Pigments and Their Synthetic Analogs
Source: Plant Cell Physiol. 2024 Aug 22;66(2):153–67. doi: 10.1093/pcp/pcae094 (PMC11879082; doi:10.1093/pcp/pcae094)
Supplement: pcae094_Supp [file pcae094_supp.zip › pcae094_Supp/Supplementary Data-1.pdf]

Supplementary Data

Chlorophyll pigments and their synthetic analogs

Hitoshi Tamiaki* and Saki Kichishima

Graduate School of Life Sciences, Ritsumeikan University, Kusatsu, Shiga 525-8577, Japan

* Corresponding author. E-mail address: tamiaki@fc.ritsumei.ac.jp (H. Tamiaki).

Table of contents

Fig. S1. Pyrrole with α/β-positions and Chl-*a* with α/β-ligations. S2

Fig. S2. Epimerization in PChlide-*a*. S2

Fig. S3. Hydrogenation of PChlide-*a* to Chlide-*a* by DPOR. S3

Fig. S4. Synthetic pathways for chemical modification of peripheral substituents in porphyrinoids. S3

Fig. S5. Synthetic pathways to **6a**–**h** with an electron-withdrawing group at the 3-position. S4

Fig. S6. Ultraviolet and visible absorption spectra of Chls-*a*/*b*/c_1 in diethyl ether. S5

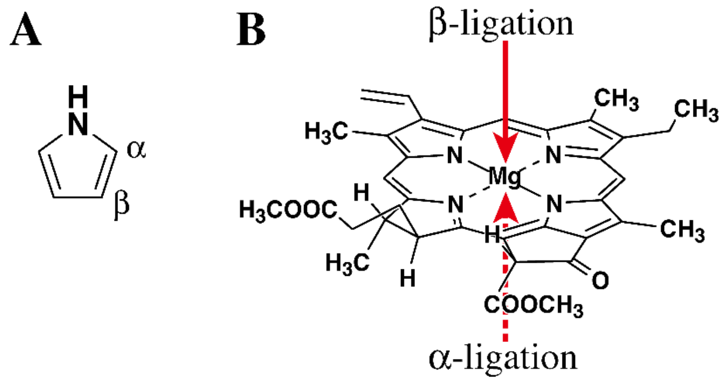

Fig. S1. Molecular structure of pyrrole with α/β-positions (A) and α/β-ligations for the axial coordination toward the central magnesium atom in Chl-*a* (B).

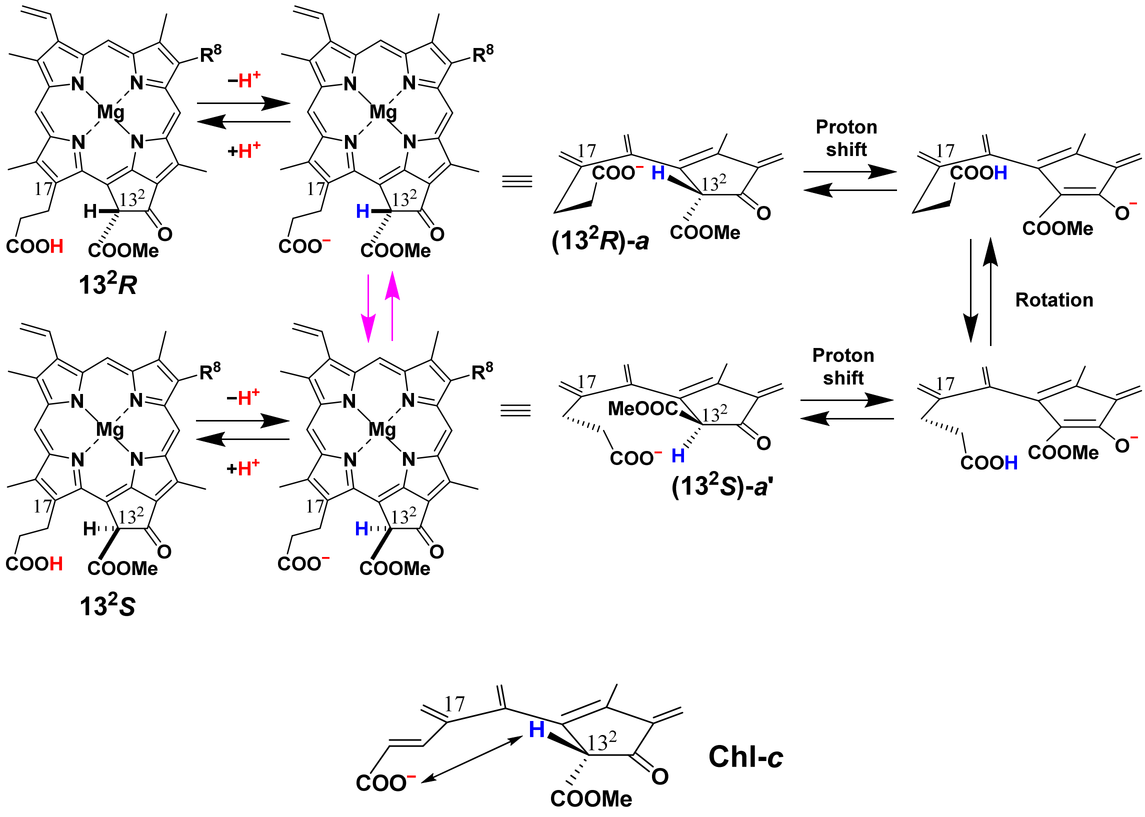

Fig. S2. Intramolecular epimerization (racemization) between (13^2*R*)-(DV-)PChlide-*a* and (13^2*S*)-(DV-)PChlide-*a′* (upper) and partial molecular structure of (13^2*R*)-Chl-*c* (lower).

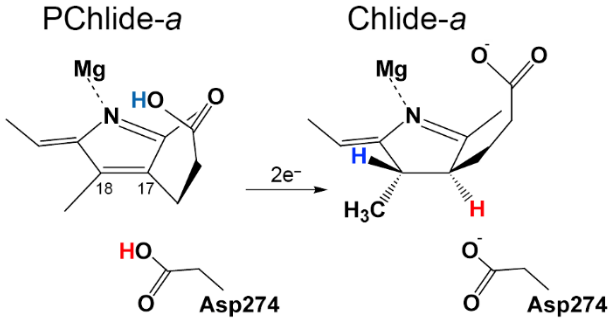

Fig. S3. Hydrogenation of PChlide-*a* (left) to Chlide-*a* (right) by DPOR.

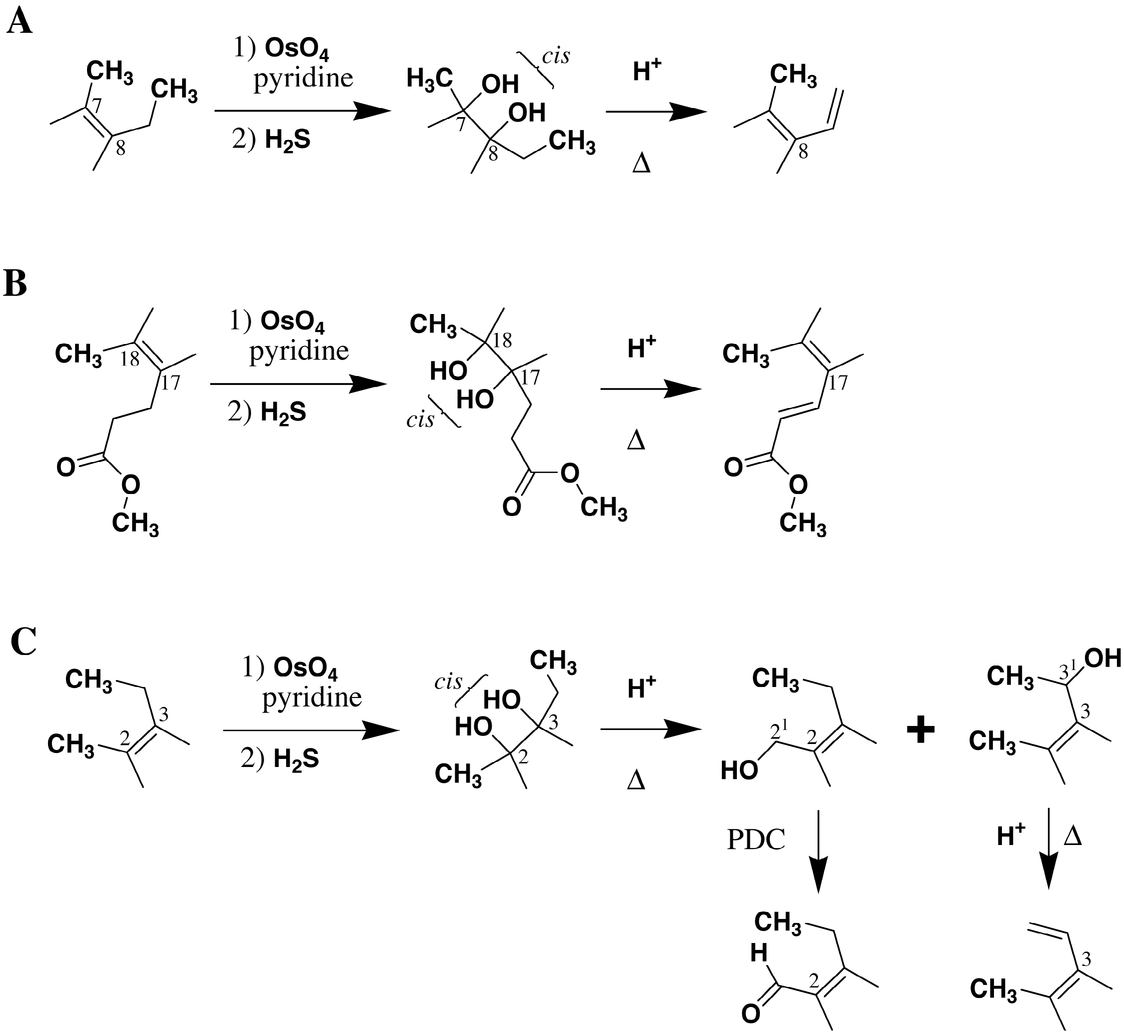

Fig. S4. *cis*-Dihydroxylation at the C7=C8 (A), C17=C18 (B), and C2=C3 double bonds (C) and dehydration at the resulting *cis*-diols to access 8-vinyl-chlorin, porphyrin with the 17-acrylate residue, and 2-formyl-chlorin, respectively.

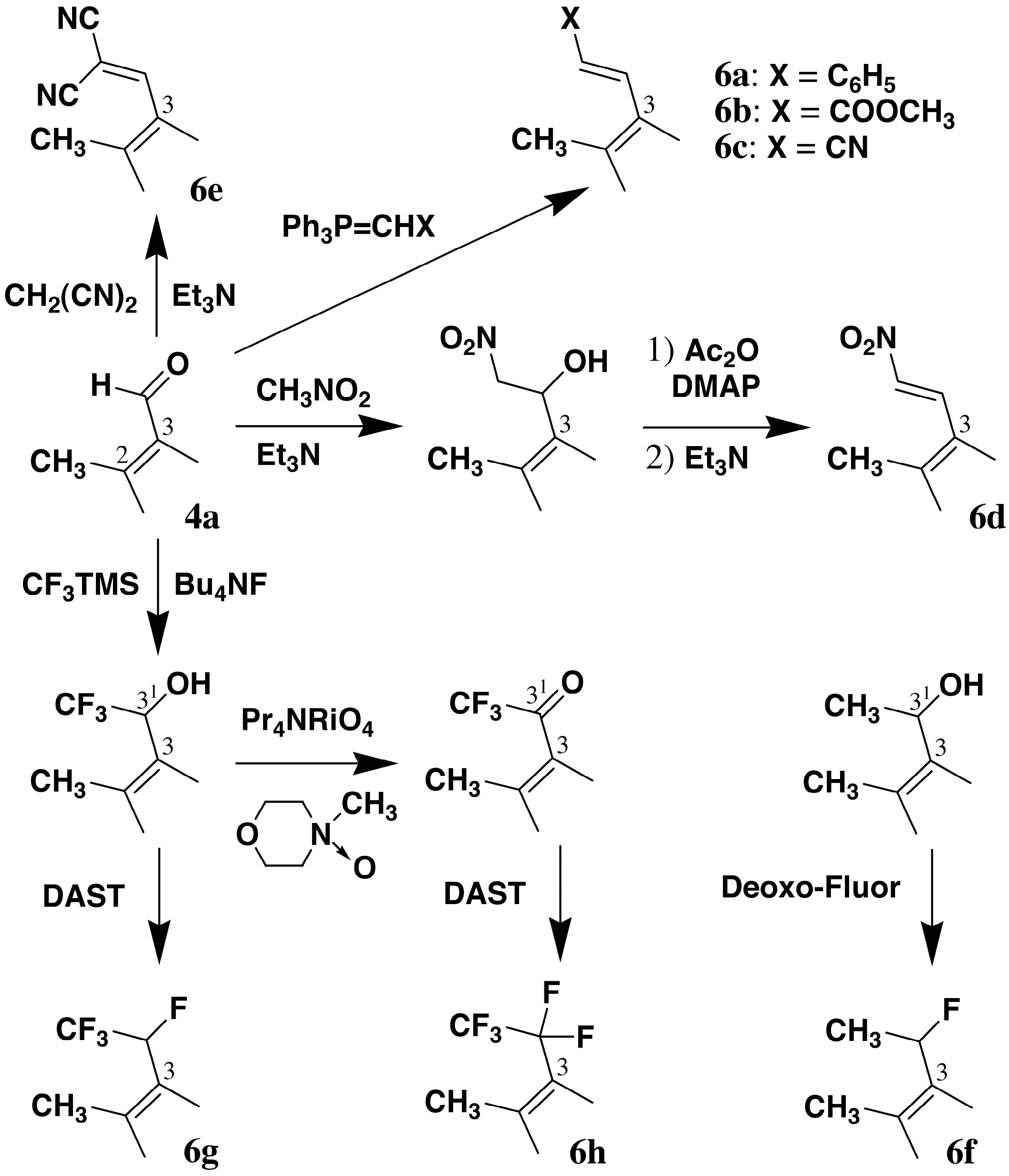

Fig. S5. Preparation of methyl 3-substituted pyropheophorbides **6a**–**h**.

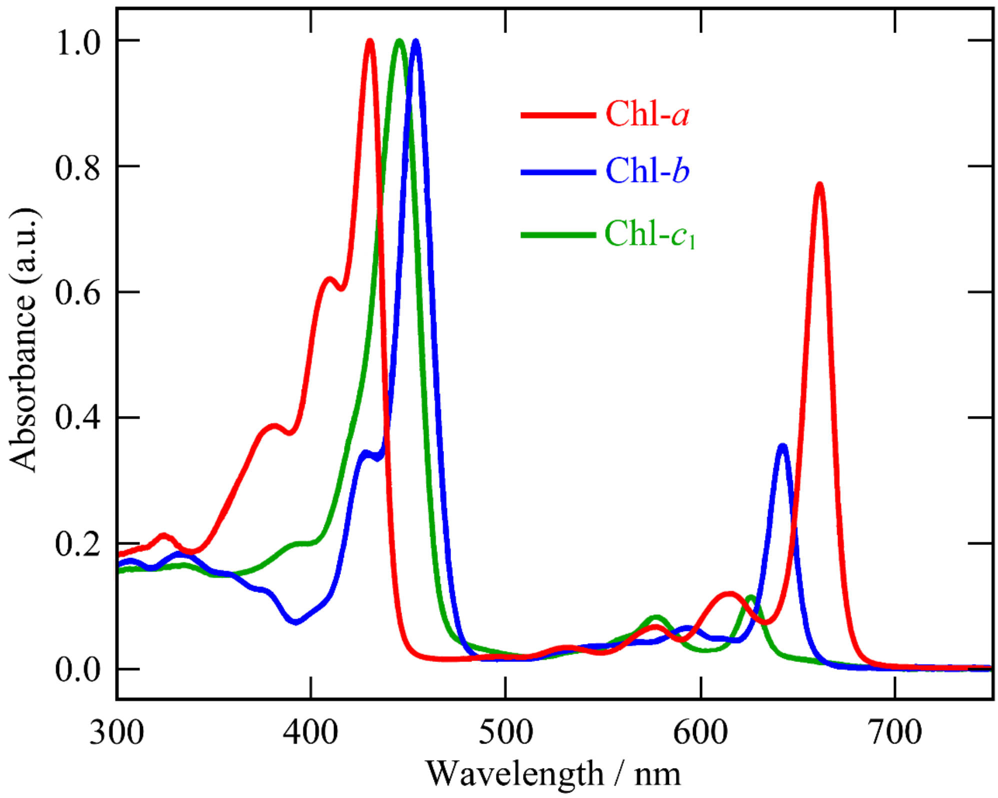

Fig. S6. Ultraviolet and visible absorption spectra of Chls-*a*/*b*/c_1 in diethyl ether: normalized at Soret maxima.
